# “All in one” nanoprobe Au-TTF-1 for target FL/CT bioimaging, machine learning technology and imaging-guided photothermal therapy against lung adenocarcinoma

**DOI:** 10.1186/s12951-023-02280-9

**Published:** 2024-01-06

**Authors:** Zhe Yang, Yujia Zhang, Lu Tang, Xiao Yang, Lei Song, Chun Shen, Andrei V. Zvyagin, Yang Li, Bai Yang, Quan Lin

**Affiliations:** 1 State Key Laboratory of Supramolecular Structure and Material, College of Chemistry, Jilin University, Changchun, 130012 China; 2 Department of Respiratory Medicine, The First Hospital of Jilin University, Changchun, 130021 China; 3 Department of Breast, China-Japan Union Hospital of Jilin University, Changchun, 130031 China; 4 College of Computer Science and Technology Jilin University, Changchun, 130012 China; 5 Australian Research Council Centre of Excellence for Nanoscale Biophotonics, Macquarie University, Sydney, NSW 2109 Australia

**Keywords:** Nanoprobe, Bioimaging-guided therapy, Machine learning, Dual-mode bioimaging, Lung adenocarcinoma

## Abstract

The accurate preoperative diagnosis and tracking of lung adenocarcinoma is hindered by non-targeting and diffusion of dyes used for marking tumors. Hence, there is an urgent need to develop a practical nanoprobe for tracing lung adenocarcinoma precisely even treating them noninvasively. Herein, Gold nanoclusters (AuNCs) conjugate with thyroid transcription factor-1 (TTF-1) antibody, then multifunctional nanoprobe Au-TTF-1 is designed and synthesized, which underscores the paramount importance of advancing the machine learning diagnosis and bioimaging-guided treatment of lung adenocarcinoma. Bright fluorescence (FL) and strong CT signal of Au-TTF-1 set the stage for tracking. Furthermore, the high specificity of TTF-1 antibody facilitates selective targeting of lung adenocarcinoma cells as compared to common lung epithelial cells, so machine learning software Lung adenocarcinoma auxiliary detection system was designed, which combined with Au-TTF-1 to assist the intelligent recognition of lung adenocarcinoma jointly. Besides, Au-TTF-1 not only contributes to intuitive and targeted visualization, but also guides the following noninvasive photothermal treatment. The boundaries of tumor are light up by Au-TTF-1 for navigation, it penetrates into tumor and implements noninvasive photothermal treatment, resulting in ablating tumors in vivo locally. Above all, Au-TTF-1 serves as a key platform for target bio-imaging navigation, machine learning diagnosis and synergistic PTT as a single nanoprobe, which demonstrates attractive performance on lung adenocarcinoma.

**Graphical Abstract:**

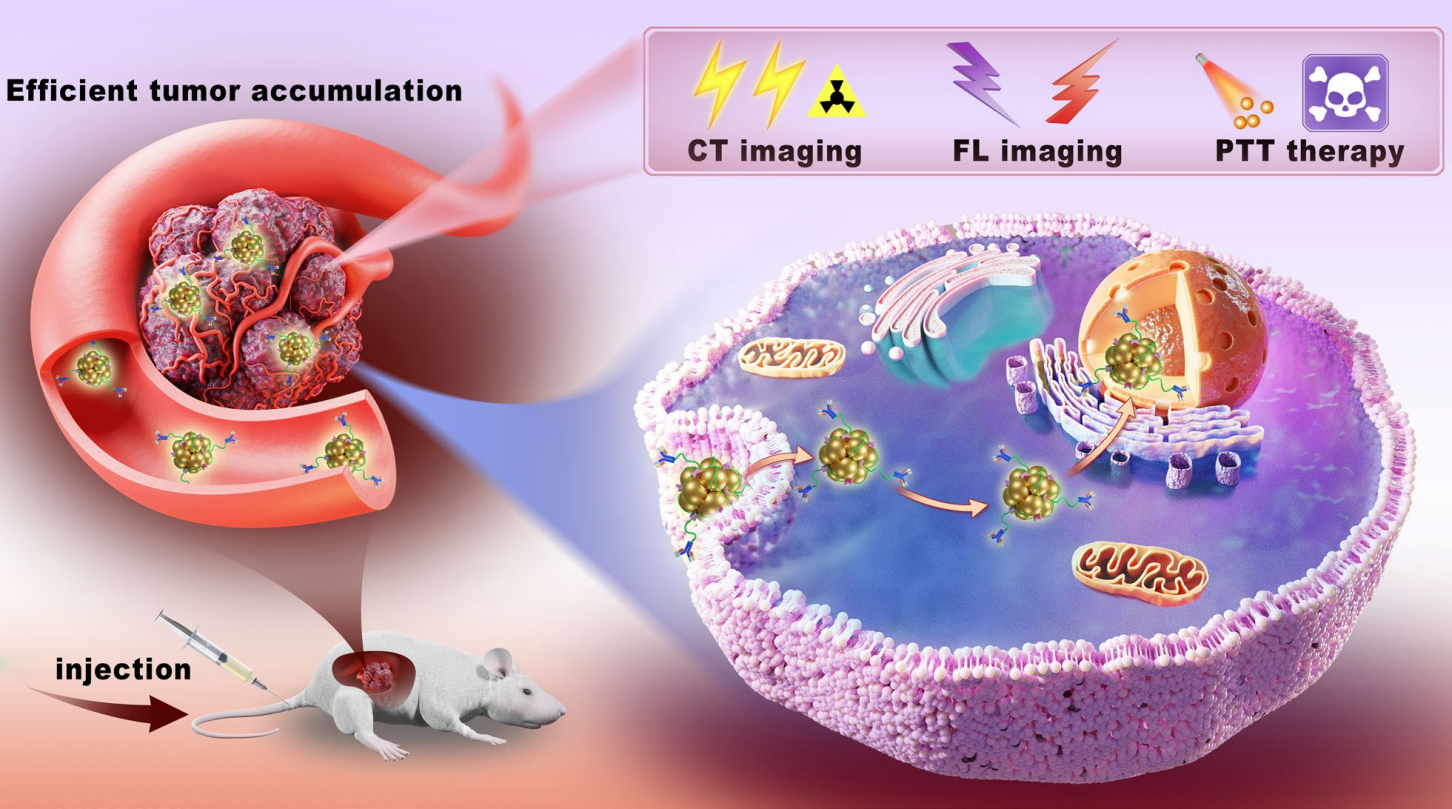

**Supplementary Information:**

The online version contains supplementary material available at 10.1186/s12951-023-02280-9.

## Introduction

The incidence of lung adenocarcinoma has been increasing in recent years, making it the most aggressive and rapidly fatal type of lung cancer. [1–3] Heterogeneous and infiltrating characteristics of adenocarcinoma hamper its accurate diagnosis and resection, leading to high mortality rates. [4–6] The main clinical treatments of cancer include surgery, chemotherapy and radiotherapy, of which surgery is the most effective approach. [7, 8] However, complete removal of tumor tissue is mandatory while remaining a challenge, as tumor-positive resection margins occur in 8–70% [9, 10]. Early detection and accurate diagnosis can greatly improve the treatment effect.

Early detection and diagnosis require materials with high sensitivity, high accuracy, and high image quality for magnification. Under such demand, the development of machine learning system software supporting the detection of lung adenocarcinoma is of great significance. With the development of digital image processing technology and deep learning, computer-aided systems play an important role in pathological section analysis to assist doctors in diagnosis and early cancer screening [11–13]. The combination of deep learning technology and highly differentiated detection probe make rapid judgment on complex cytopathological images, which is low cost, fast, and free from the influence of experience [9, 14, 15]. Machine learning assists in expert diagnosis, reduces the subjectivity of diagnostic results and improvesdiagnostic efficiency.

In addition to early diagnosis before surgery, intraoperative imaging also requires targeted and high differentiation of fluorescent probes. Intraoperative guidance is necessary for surgeons to preserve as much healthy tissue as possible. Current visual based on white light and tactile guidance methods are insufficient [16–18], thus exploration of real-time intraoperative guidance techniques for complete and safe tumor resection is of great significance.

FL imaging has shown potential for guiding surgeons during complex interventions as a non-invasive tool, given its high spatial resolution and instantaneity [19–21]. Clinical useable fluorescent agents like indocyanine green (ICG) and methylene blue (MB) have been extensively explored for a variety of tumor margin delineation [22, 23]. However, short time of tumor retention, photobleaching, rapid systematic clearance, and lack of target properties have impended FL imaging-guided tumor treatment. [24–26] Besides, commonly used fluorescent probes including quantum dots (QDs), organic dyes, and single-walled carbon nanotubes (SWCNTs), are often unsuitable for long-term in vivo fluorescent imaging [27–29], on accounting of inherent toxicity, short blood half-lives or poor biocompatibility. [30, 31] Novel fluorescent probes with high FL stability and target properties are needed for imaging-guided treatment of lung adenocarcinoma.

Interestingly, AuNCs as a few nanomaterials proven by the Food and Drug Administration (FDA), have gained significant attention because of their good optical properties. [32] Owing to their ultra-small size, they have excellent FL properties and good biocompatibility. Besides, AuNCs perform outstanding CT imaging properties, for their attenuation coefficient of X-rays is higher than commonly used lipiodol (at 100 keV, Au 5.16 cm^2^/g, I 1.94 cm^2^/g) [33]. Importantly, AuNCs are promising photothermal therapy agents with high light-to-heat conversion efficiency for further treatment of lung adenocarcinoma [34–36]. Compared with conventional chemotherapy and radiotherapy, photothermal therapy (PTT) has many advantages in local tumor treatment, such as a minimally invasive process, highly controllable operation, and fewer side effects during the external and remote stimulus of local heating [37–39]. Further, targeted imaging of lung adenocarcinoma is expected to give full play to the advantages of PTT leading to noninvasive treatment. It is reported that more than 85% of lung adenocarcinoma expressTTF-1 [40]. Due to its high expression, high specificity and strong sensitivity, TTF-1 is a promising biomarker for tracking lung adenocarcinoma [41].

In this work, multifunctional nanoprobe Au-TTF-1 and machine learning software Lung adenocarcinoma auxiliary detection system are designed and synthesized, which demonstrates the paramount importance of nanoprobe based on diagnosis and bioimaging-guided treatment of lung adenocarcinoma. TTF-1 antibody combined with dual-mode imaging AuNCs to form nanoprobe Au-TTF-1 through coupling reaction, which specifically recognized the highly expressed TTF-1 antigen in lung adenocarcinoma and light up lung adenocarcinoma cells with bright red FL. The FL imaging intensity of cancer cells and normal cells is significantly different, and machine learning software introduced Elliptic Fourier descriptors for the first time to represent the boundary contour and position parameters of individual cells in the image innovatively, which clearly distinguished suspected cancerous cells from normal cells. They performed excellent FL and CT properties for targeting precise positioning of the lung adenocarcinoma before treatment, especially for the clear distinction between tumor and normal tissues. Au-TTF-1 penetrates into tumor, and further implements photothermal treatment noninvasively, resulting in ablating tumors in vivo locally. Au-TTF-1 integrated machine learning imaging and targeted PTT in a single prospective nanoprobe, achieving detection and noninvasive treatment navigation, which exhibited satisfactory results against lung adenocarcinoma.

## Experimental section

### Preparation of Au-TTF-1

First, 1 mL Tween 20 was added into 1 L PBS solution to prepare TPBS solution. Then 10 μL TTF-1 antibody with a mass concentration of 1 mg/mL was added to 240 μL TPBS for dilution. Next, Freeze-dried AuNCs of 100 mg were weighed, and dissolved with 10 ml TPBS to prepare AuNCs solution with a mass concentration of 10 mg/ mL. Finally, 250 μL of diluted TTF-1 antibody was added with 7.2 mg EDC and 2.4 mg NHS for activation. After stirring at room temperature for 30 min, 2 mL of AuNCs solution was added and placed in a cold chamber at 4 °C for reaction with stirring for 8 h. The outcome Au-TTF-1 aqueous solution was freeze-dried and stored at −20 °C temperature for later use.

### External flow cytometry and FL imaging

A549 and Beas-2B cells were seeded in 6-well plate culture dishes at a seeding density of 2 × 10^5^ cells/well and incubated for 24 h under culture conditions. Cells were digested with trypsin (Hyclone, Logan, UT, USA), washed three times with PBS, and resuspended with 0.3 mL of 1 × loading buffer. The FL intensity of cells incubated without AuNCs was gated as baseline FL. Three independent experiments were carried out to analyze and compare the percentage of fluorescent-positive cells in each group.

A549 and Beas-2B cells were seeded in 6-well plate culture dishes at a seeding density of 2 × 10^5^ cells/well and incubated for 24 h under culture conditions. After incubation, the cells were washed twice with PBS and then treated with Au-TTF-1 at a concentration of 300 μg/mL for 6 h. After discarding the nanoclusters, the samples were washed three times with PBS, fixed with paraformaldehyde for 30 min, and then sealed with anti-fluorescence attenuation mounting medium containing DAPI for laser scanning confocal test.

### Design of Lung adenocarcinoma auxiliary detection system

The implementation of the algorithms in this chapter is based on the common Pytorch framework. The training, verification, and testing process is carried out on an Ubuntu NVidia GeForce GTX 2080 Ti GPU. During training, the optimizer selects Adam. In addition, the experiment set the initial learning rate to 10e^−4^, weight attenuation to 10e^−8^, batch size to 256, and iteration number to 90 epochs. In the training process, the network input image is first normalized to the gray level, then randomly cropped to 256 × 256 size, and the data is enhanced by random horizontal and vertical flipping. The center coordinates of the contour and the elliptic Fourier descriptor parameters are obtained by using Fourier transform for the real mask label.

### In Vitro* photothermal therapy evaluations*

A549 cells were seeded in 6-well plate culture dishes at a seeding density of 2 × 10^5^ cells/well and incubated for 24 h under culture conditions. After incubation, the cells were washed twice with PBS and then treated with Au-TTF-1 at a concentration of 300 μg/mL for 6 h. Then the medium was irradiated with a 2 W cm^−2^ laser at 0 min, 1 min, 5 min, and 10 min. Next, cells were digested with trypsin (Hyclone, Logan, UT, USA), washed three times with PBS, and resuspended with 0.3 mL of 1 × loading buffer. Finally, the cells were stained by Annexin V/PI and detected apoptosis at different points by flow cytometry.

### Evaluation of tumor-bearing mouse model and FL/CT Imaging

Male C57 mice (6–8 weeks old) were purchased from the Laboratory Animal Center of the First Hospital of Jilin University (Jilin, China), and all animal experimental protocols were approved by the Ethical Committee of the First Hospital of Jilin University. The tumor-bearing mouse model was established, and 1 × 10^6^ Lewis cells were injected subcutaneously into the axilla of each mouse. The experimental subjects were Lewis tumor-bearing mice with a tumor volume of about 1500 mm^3^, which was approved by the Experimental Animal Center of the Institute of Translational Medicine, The First Hospital of Jilin University.

For in vivo FI/CT imaging, 200 μL of Au-TTF-1 was injected via tail vein into each Lewis tumor-bearing mouse (n = 3). Then images were collected at different time points before (0 h) and after (2 h, 4 h, 6 h, 6.5 h, 7 h, 12 h, 24 h) injection. The relative imaging intensity of the tumor area was measured and expressed as mean SD.

### In vivo* photothermal therapy evaluations and biocompatibility Analysis*

One week after tumor bearing, the tumor grew to approximately 120 mm^3^, which was considered as successful tumor growth. 200 μL of 300 μg/mL Au-TTF-1 was injected through the tail vein into the mice (n = 6), NIR therapy was performed after 6 h, the treatment was carried out at 0, 3, 6, 9 and 12 days. The tumor volume was first recorded and calculated using the following formula: V(mm^3^) = (L × S^2^)/2. Where L is the largest tumor diameter (mm) and S is the smallest tumor diameter (mm). Then 200 μL of 300 μg/mL (~ 3 mg/kg) Au-TTF-1 was injected through the tail vein into the mice, and the internal temperature was monitored every minute by infrared thermal imaging after 6 h. Two weeks after the fifth treatment, all animals were sacrificed, and tumor tissues and major organs were collected. H&E staining was used for pathological examination. TUNEL and PCNA immunohistochemistry were used to detect the apoptosis and proliferation of tumor cells.

To evaluate the biocompatibility of Au-TTF-1 in vivo, all C57 mice were anesthetized and euthanized (n = 6). For histological examination, the organs of the heart, liver, spleen, lung, and kidney sections were stained with eosin (H&E), and then the pathological changes were observed by light microscopy.

### statistical analyses

The experimental data were analyzed by GraphPad Prism 7.00 software. The experimental results were represented by mean ± standard deviation, comparison between two groups was performed by t-test, and comparison between multiple groups was performed by ANOVA analysis.

## Results and discussion

### Design principle and synthesis strategy of Au-TTF-1

**Scheme 1:**
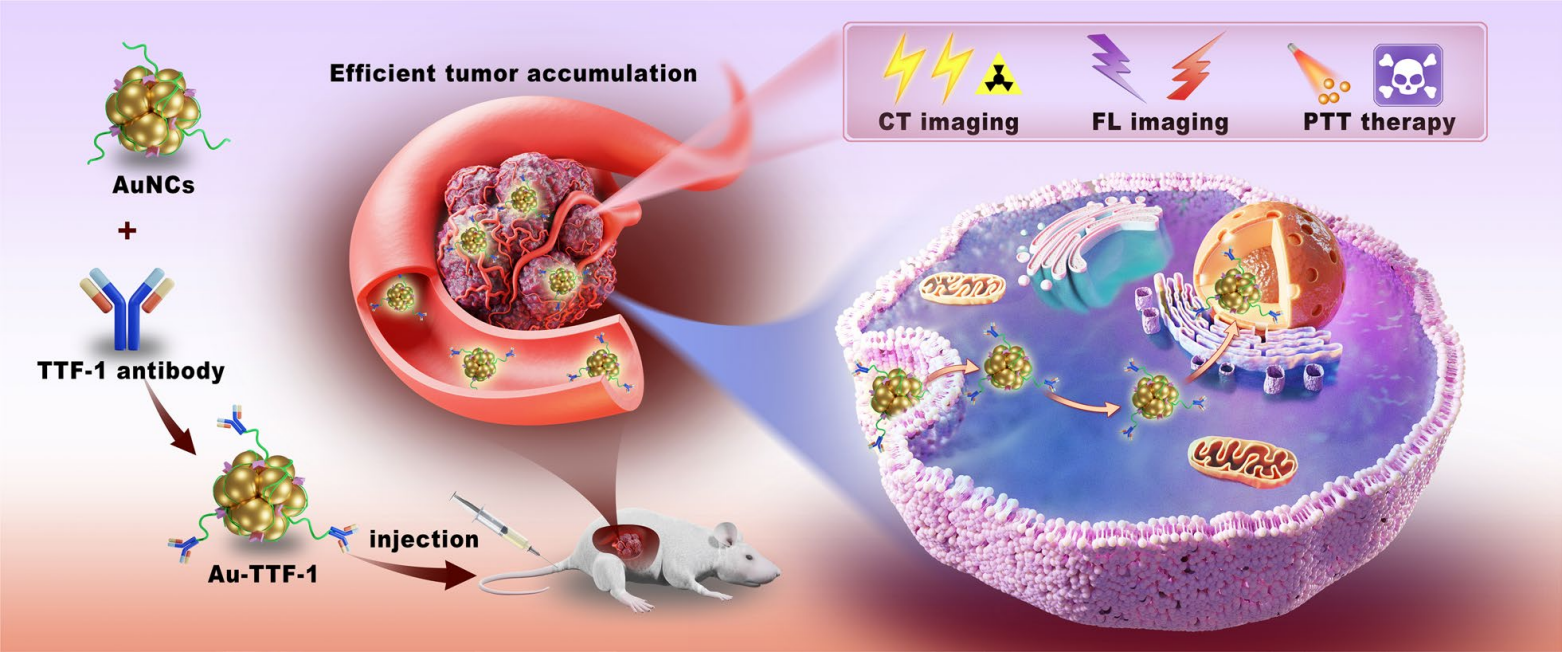
Illustration of Au-TTF-1 in preparation and efficient tumor accumulation for target CT/FL imaging as well as photothermal therapy (PTT) against tumor

The design strategy and preparation of Au-TTF-1 were shown in Scheme 1. AuNCs combined with TTF-1 antibody to form multifunctional nanoprobe Au-TTF-1. Au-TTF-1 specifically targeted to lung adenocarcinoma cells, which integrated machine learning diagnosis, target CT/FL imaging and imaging-guided PTT in a single prospective nanoprobe, achieving detection and noninvasive treatment navigation.

**Fig. 1:**
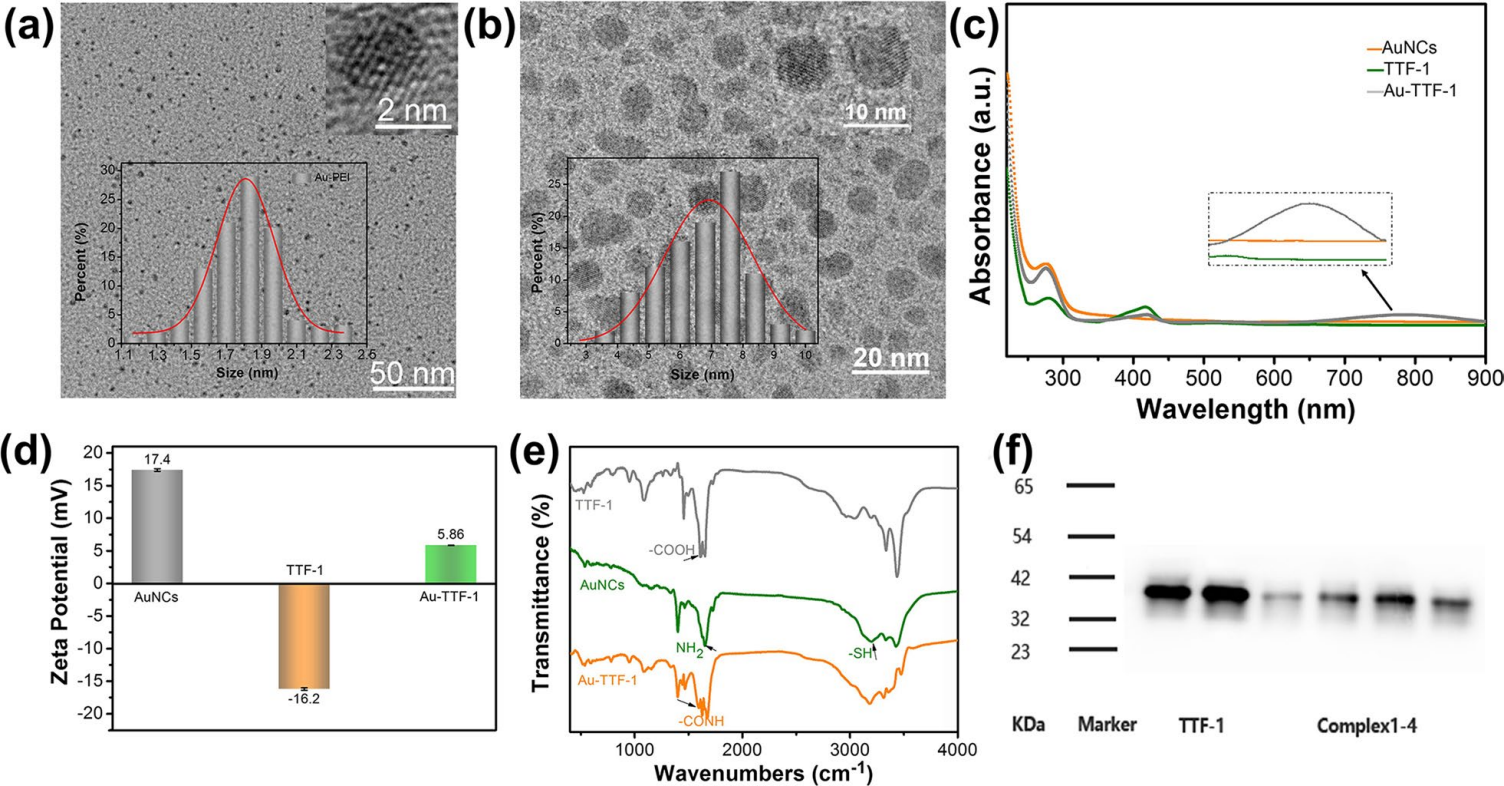
Synthesis and characterization of Au-TTF-1. Morphology and microstructure image of **a** AuNCs and **b** Au-TTF-1, inset was the HRTEM and the relative size distribution histogram based on 100 nanoparticles. **c** UV absorbance, **d** Zeta potential, **e** FTIR and **f** Gel electrophoresis patterns of AuNCs, TTF-1 and Au-TTF-1

First, the morphology, size, distribution, and microstructure of AuNCs as well as Au-TTF-1 were characterized. As shown in Fig. 1a, b and Additional file 1: Fig. S1, the size of the nanoprobe increased from 1.8 to 6.7 nm, and the change trend of hydrodynamic radius was consistent, which initially indicated the occurrence of the coupling reaction. In Fig. 1c, UV–Vis spectra displayed that Au-TTF-1 possessed characteristic peaks at 279 nm and 413 nm, which combined the absorbance of AuNCs and TTF-1. This certified the successful connection. Besides, a wide UV absorption peak appeared around 790 nm, which was conducive to photothermal treatment. The charge also changed after the coupling reaction in Fig. 1d, AuNCs displayed a positive charge of + 17.4 mV because of the plenty –NH_2_ groups from polyvinylimine ligand, and the Au-TTF-1 nanoprobe had an intermediate charge of + 5.86 mV. Since the phospholipid groups on the cell membrane had negative charge, nanoparticles with appropriate positive charge were more easily taken up by the cells. Further, the FTIR spectrum in Fig. 1e revealed the reaction between the TTF-1 and AuNCs, which was associated with the gel electrophoresis image in Fig. 1f for the molecular weight of the complex is about 40 kDa. These results all verified the successful synthesis of well-designed nanoprobe Au-TTF-1.

### FI/CT Imaging and photothermal properties of Au-TTF-1

**Fig. 2:**
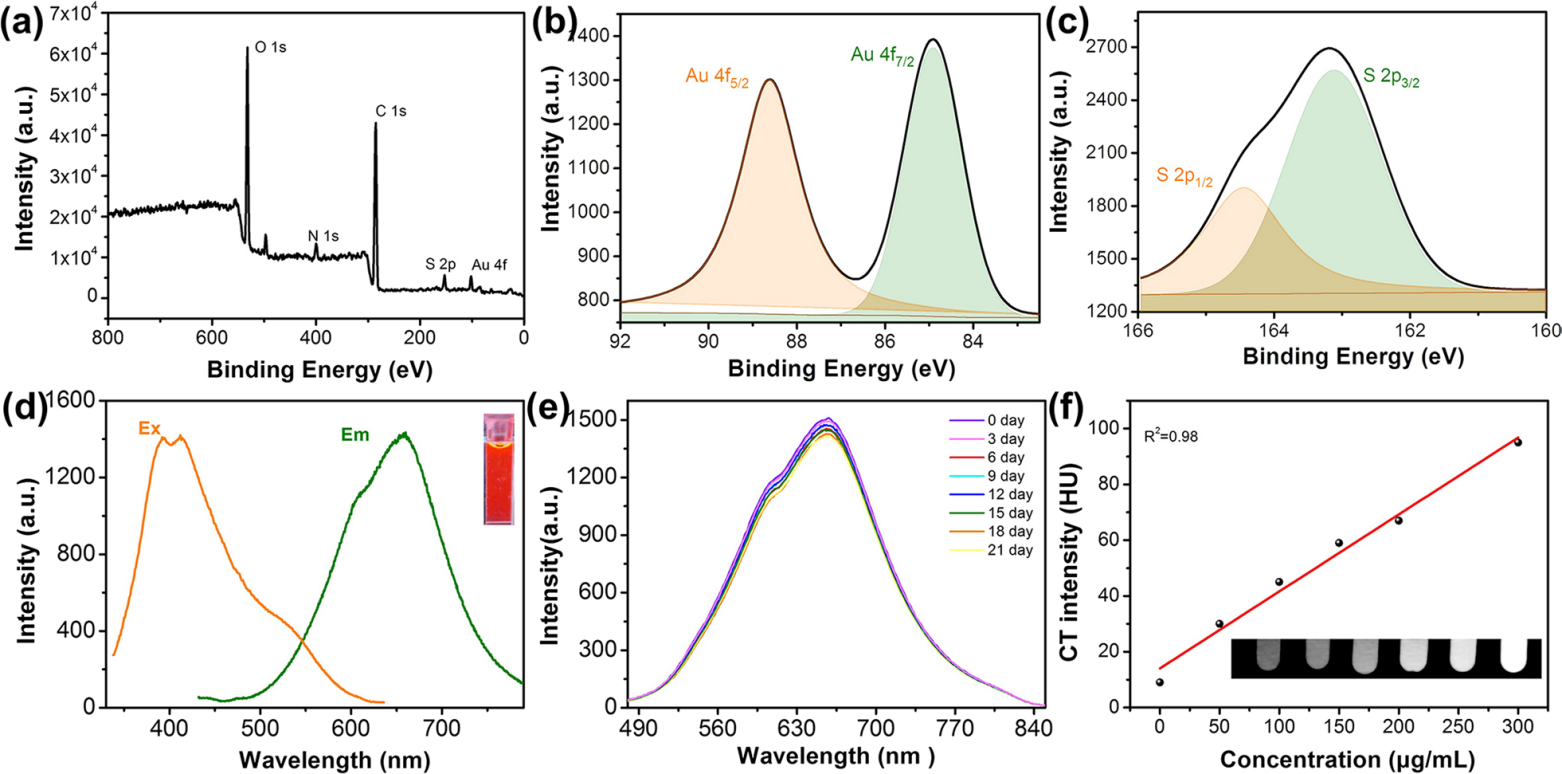
XPS spectrum (**a**) and high-resolution XPS spectra of **b** Au 4f and **c** S 2p in the fabricated Au-TTF-1. **d** Excitation and emission spectra of Au-TTF-1. **e** The optical stability of the fluorescent Au-TTF-1 in 21 days. **f** CT images and intensities of Au-TTF-1 with gradient concentration of 0, 50 μg/mL, 100 μg/mL, 150 μg/mL, 200 μg/mL and 300 μg/mL (R^2^ = 0.98)

After the successful connection, the optical properties of the Au-TTF-1 were further verified. XPS spectrum (Fig. 2a,b) indicated the electronic binding energies of Au 4f were located at 88.2 eV and 84.3 eV, corresponding to Au(0) and Au(I), respectively. The former state was good for nucleation, and the latter state was easy to combine with the ligand stably, which formed a metal-sulfhydryl covalent bond in Fig. 2c. The FL emission wavelength of the prepared Au-TTF-1 was located at 650 nm in Fig. 2d, red emission is advantageous to penetrate tissues deeper and distinguish from autogenous blue FL. In Fig. 2e and Additional file 1: Fig. S2, Au-TTF-1 exhibited photophysical properties in favor of long-term biomedical imaging. They have bright red FL with excellent stability in terms of time, pH, photobleaching resistance, and solvent. The outstanding FL stability is very beneficial for long-term in vivo imaging. Besides, as a dual-mode imaging contrast agent, the CT intensity of Au-TTF-1 was up to 95 HU when the concentration was 300 μg/mL (Fig. 2f), meeting the needs of in vivo imaging. When the concentrations of Au-TTF-1 increased (from left to right), the corresponding imaging intensities grew proportionally, there was a good linear relationship between the concentrations and CT intensities (R^2^ = 0.98). Therefore, the Au-TTF-1 can be traced in vivo through high and quantitative imaging intensities.

**Fig. 3:**
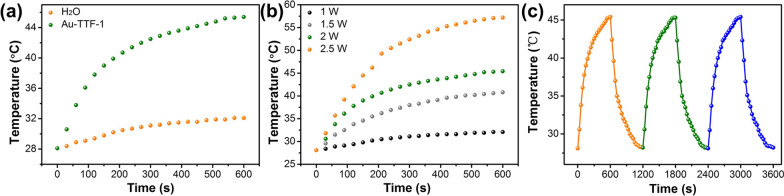
PTT performance of Au-TTF-1. **a** Photothermal heating curves with Au-TTF-1 and H_2_O upon 808 nm laser irradiation (2.0 W cm^−2^). **b** Photothermal heating curves with different irradiation wattage values from 1 to 2.5 W cm^−2^. **c** Temperature elevation of the 300 μg/mL Au-TTF-1 under 2.0 W cm^−2^ irradiation over three laser on/off cycles

As a PTA, the photothermal properties of Au-TTF-1 were further evaluated. First, the heating curves of Au-TTF-1 were monitored. Temperature elevation of Au-TTF-1 quickly increased and finally reached 17.1 °C, whereas the temperature of the water as a control increased only 4.3 °C (Fig. 3a). Afterward, in Fig. 3b, the temperature of the Au-TTF-1 with 1 W cm^−2^ did not change significantly, while laser irradiation with 1.5, 2, and 2.5 W cm^−2^ led to a marked increase from room temperature 28 to 40.8, 45.4, and 57.2 °C in 600 s, respectively. It was confirmed that modest temperatures led to a more resultful treatment in the local tumor cells, [35] which guaranteed the safety of PTT. Thus, 300 μg/mL concentration of Au-TTF-1 and 2 W cm^−2^ laser power for 600 s were employed for the following experiments, which meet the requirements for cancer cell damage. Furthermore, The thermal cycling performance of Au-TTF-1 was tested by recording the temperature with irradiation and then cooled to room temperature naturally three times. As revealed in Fig. 3c, the temperature variation of each cycle was almost identical. These results indicated that Au-TTF-1 as a PTA had the potential to cure lung adenocarcinoma.

### In vitro* imaging, machine learning diagnosis and target therapy effect of Au-TTF-1*

**Fig. 4:**
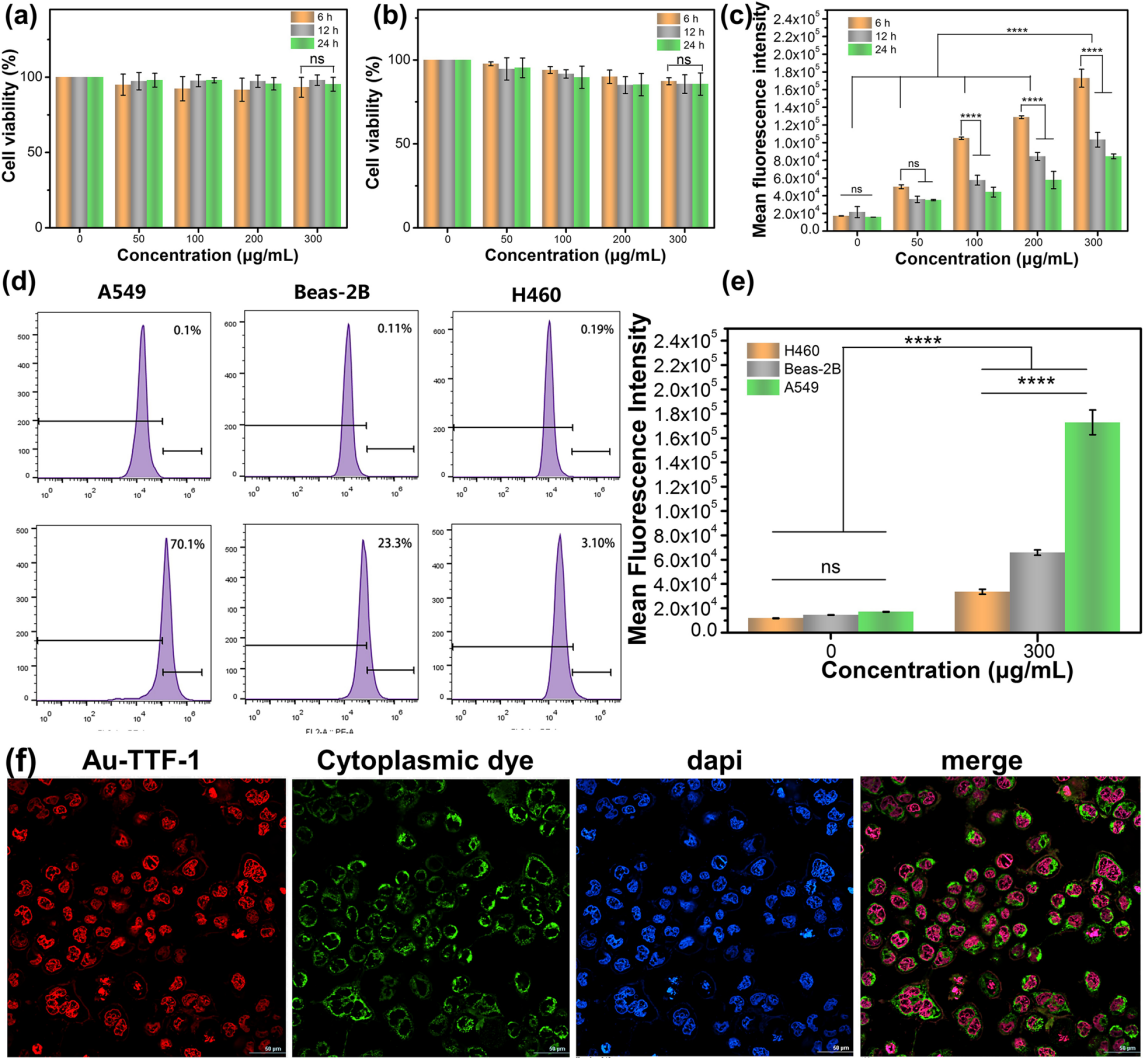
**a** CCK-8 test of A549 cells cultured with gradient concentration Au-TTF-1 for 6 h,12 h and 48 h. **b** CCK-8 test of Beas-2b cells cultured with gradient concentration Au-TTF-1 for 6 h,12 h and 48 h. (**c**) Flow cytometry analysis of cellular uptake and the quantified mean FL intensity of A549 cells cultured with gradient concentration Au-TTF-1 for 6 h, 12 h and 24 h. (**d**) Flow cytometry analysis of cellular uptake and **e** the Quantified mean FL intensity of A549 cells, Beas-2B cells and H460 cells cultured with 300 μg/mL Au-TTF-1 for 6 h. **f** Laser scanning confocal micrographs of intracellular distribution in A549 cells after culturing with Au-TTF-1, MitoTracker dyes and dapi, All scale bars are 50 μm

After demonstrating the optical properties of Au-TTF-1, cellular experiments were performed to explore the biocompatibility and target therapeutic effect on lung adenocarcinoma. The cytotoxicity of Au-TTF-1 was evaluated by co-culture with A549 cells and Beas-2B at the series of concentrations (0, 50, 100, 200 and 300 μg/mL) for 6 h, 12 h and 24 h, respectively. The results in Fig. 4a and Fig. 4b showed that Au-TTF-1 had biocompatibility. Au-TTF-1 was highly ingested and endocytosed, exhibiting high FL intensity in Fig. 4c. The positive charge of Au-TTF-1 is also attributed to combining with the phospholipid bilayer (negative charge) on the cell membrane. Further, in Fig. 4d and Additional file 1: Fig. S3, flow cytometry of Au-TTF-1 cultured with A549, Beas-2B and H460 were performed to verify the specific targeting ability at the cellular level quantitatively. The quantitative analyses of the FL intensity in Fig. 4e then showed that the A549 cells had more apparent red FL intensity (1.7 × 10^5^ a.u.) compared with Beas-2B (0.65 × 10^5^ a.u.). The uptake rate of Au-TTF-1 by lung adenocarcinoma cells was three times higher than that of common lung epithelial cells. The results further demonstrated the specific binding affinity to lung adenocarcinoma of Au-TTF-1.

To further study the accurate location of the Au-TTF-1 in depth, dyes were used to stain the cytoplasmic and nucleus. As shown in the FL images (Fig. 4f), the red FL of the Au-TTF-1 was merged with the blue FL of dapi and appeared in pink color, while it did not correspond with the green FL of MitoTracker and clear boundaries were observed. In stark contrast, Au-TTF-1 barely showed red fluorescence in Beas-2B (Additional file 1: Fig. S4). This result also verified the successful connection and preparation of nanoprobe, for TTF-1 was expressed in the nucleus.

Au-TTF-1 had the advantages of high differential expression and localization in the cell nucleus, so machine learning software Lung adenocarcinoma auxiliary detection system was designed, which combined with Au-TTF-1 to assist the intelligent recognition of lung adenocarcinoma jointly. The difficulty and key technology of the auxiliary diagnosis system against lung cancer were the accurate recognition and segmentation of cells in the pathological images. In this system, elliptic Fourier descriptors were introduced to represent the boundary contour and position parameters of individual cells, and deep learning was used to design and train a cell recognition convolutional neural network based on the feature pyramid structure, which can effectively output the information of individual cells in the image.

**Fig. 5:**
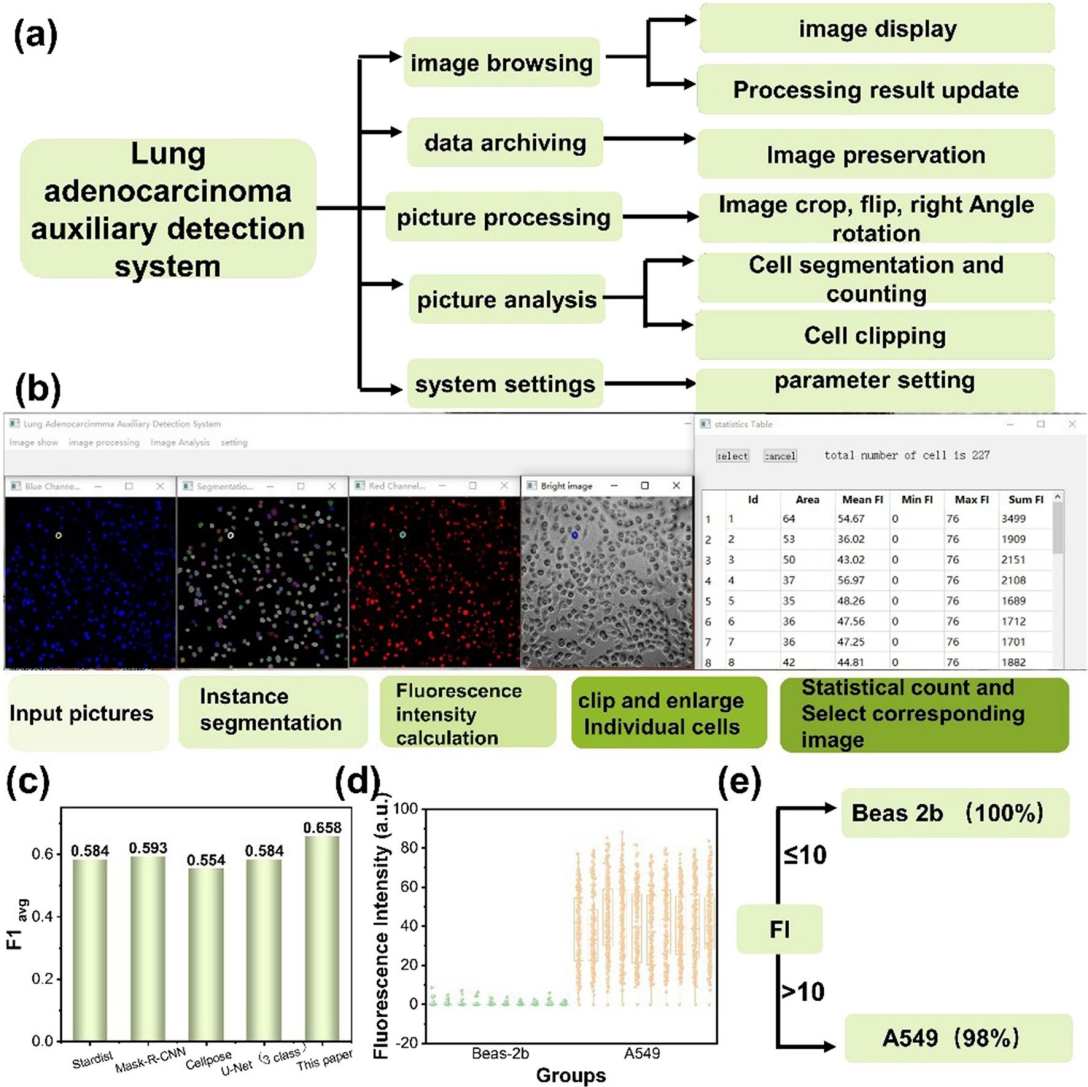
**a** Overall function of lung adenocarcinoma auxiliary detection system. **b** Demonstration of software operation steps and output results. **c** The average time of each cell segmentation method. **d** The FL intensity calculated by software detection of Beas-2b and A549 cells. **e** Counts of cells with FL intensity greater than 10 and less than 10 in A549 and Beas-2b cells

The function of the whole system is to display, view and classify the collected pathological cell images through the algorithm. Further, suspect lung adenocarcinoma cells were selected through data analysis of FL intensity distribution. The system contributed to assisting professional doctors make more accurate diagnostic assessments. The variety functions of Lung adenocarcinoma auxiliary detection system were shown in Fig. 5a, including image browsing, data archiving, image processing, image analysis, and system settings. There are corresponding operation options in the software, and specific operation process was shown in Fig. 5b and Additional file 1: Video S1. First, the dapi image of the blue channel was input to segment the cells through the contour recognition network proposed in this paper. The segmentation mask was obtained and the cell count was completed. The second step was to transform the three-channel image of Au-TTF-1 distribution into a single-channel grayscale image. The grayscale value of each pixel represented the FL intensity value. Thirdly, FL intensity value of each cell was exported, and suspicious cells were selected in batches according to the set threshold. At the same time, a single cell can be selected to crop and enlarge, and the images of each channel were saved for observation. The effectiveness of the detection system algorithm was based on the accuracy of cell recognition and boundary segmentation. The contour recognition network proposed in this paper is superior to some other proposed algorithms in segmentation index. The average F1avg of precision and recall ratio is 0.658, which is better than other methods reported (Fig. 5c).

$$F_{1} \,avg\, = \,{1 \mathord{\left/ {\vphantom {1 5}} \right. \kern-0pt} 5}\sum\nolimits_{\tau } \in \,\,T\,F_{1\,} ,\,T\, = \,\left( {0.5,\,0.6,\,0.7,\,0.8,\,0.9} \right)$$

Besides, ten groups of common lung epithelial cells Beas-2b and adenocarcinoma cells A549 were analyzed by the Lung adenocarcinoma auxiliary detection system, the data analysis was carried out and drawn as shown in Fig. 5d, e. The average FL intensity of the two cell images was significantly different. The average intensity was close to zero for Beas-2b, while the average intensity is greater than 40. Besides, the histogram divides cells into two categories according to the average FL intensity greater than 10 and less than or equal to 10. It can be seen from the data distribution that the average FL intensity of all normal cells was lower than 10, while there were only 0–4 cells with FL intensity less than 10 in each A549 cell group. Labeling images and distribution statistics obtained from Au-TTF-1 and the detection system clearly distinguished suspected cancerous cells from normal cells. Machine learning and the highly differentiated uptake of Au-TTF-1 with FL imaging played an important role in assisting the recognition of lung adenocarcinoma.

**Fig. 6:**
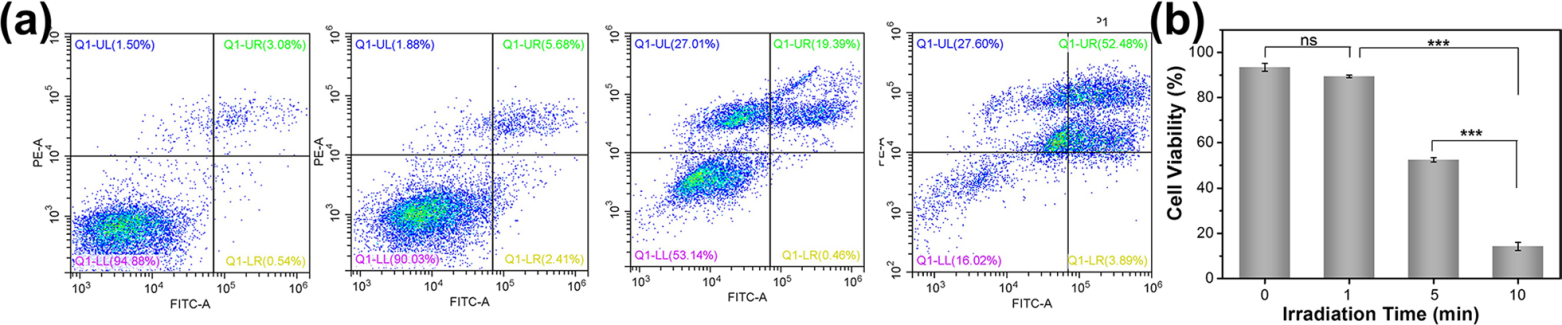
**a** Flow cytometry analysis of cell viability cultured with 300 μg/mL Au-TTF-1 irradiated with 0, 1 min, 5 min and 10 min. Q1, necrosis (up left); Q2, late apoptotic (up right); Q3, viable (low left); Q4, early apoptotic (low right). **b** Quantitative bar chart of cell activity

Based on the previous exploration of photothermal properties, A549 cells were cocultured with 300 μg/mL Au-TTF-1 for 6 h, and irradiated with 2 W cm^−2^ laser power at 0 min, 1 min, 5 min, and 10 min. In Fig. 6a, the number of early apoptosis, late apoptosis and cell death was almost negligible before irradiation, which exhibited the high bio-compatibility of Au-TTF-1 again. In Fig. 6b, the results quantificationally showed that after 600 s laser irradiation, the cells began to appear with a large number of deaths, confirming the good PTT potency in vitro.

### In vivo* FL/CT imaging and antitumor effect*

**Fig. 7:**
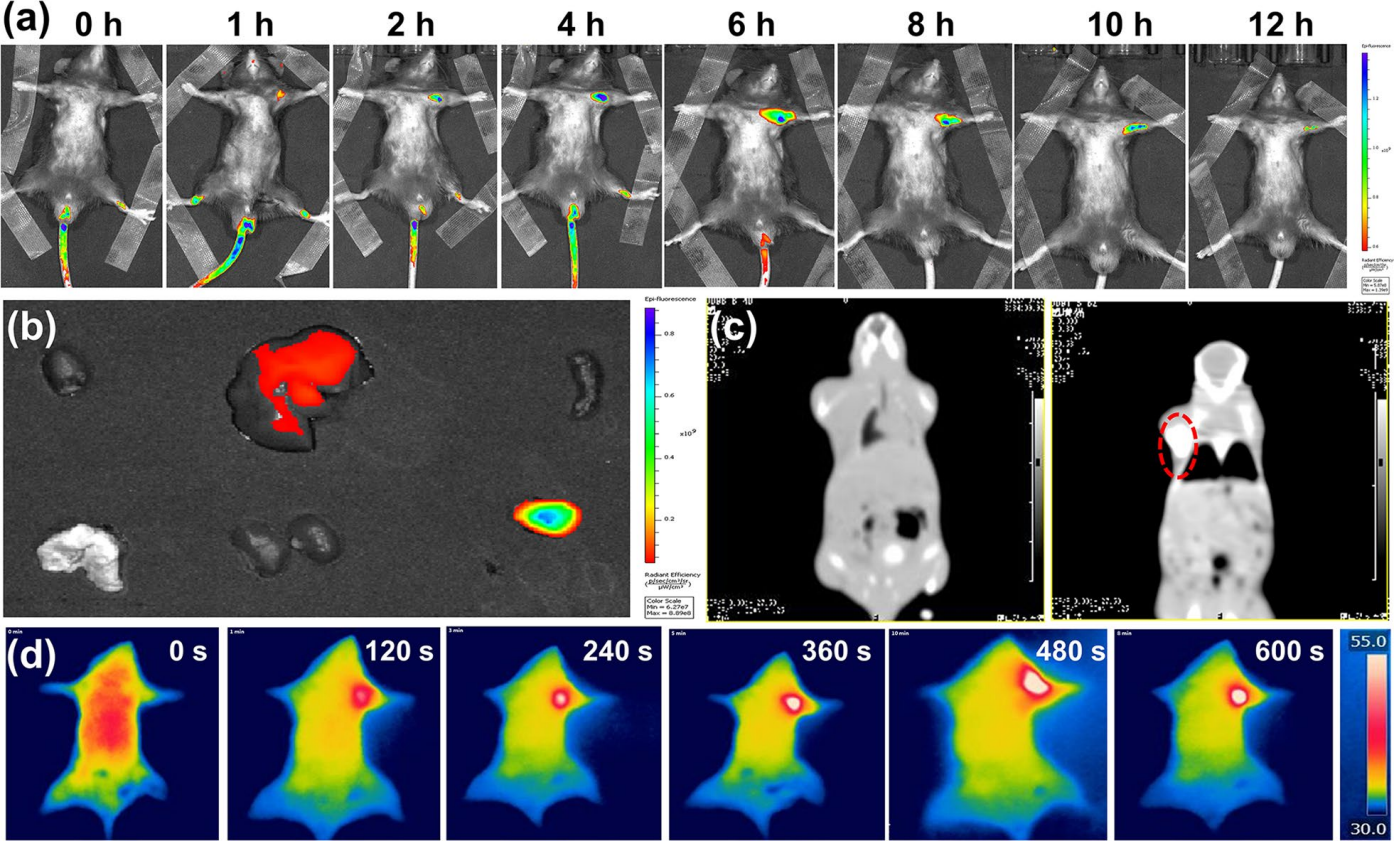
(**a**) In vivo FL images of mouse injected with Au-TTF-1 at various time points 0, 1, 2, 4, 6, 8, 10, 12 h. (b) The FL image of organs and tumor. **c** In vivo CT images of mice before and after. **d** IR images at 0, 120, 240, 360, 480, and 600 s under 2.0 W cm^−2^ irradiation after intravenously injected Au-TTF-1 for 6 h

**Fig. 8:**
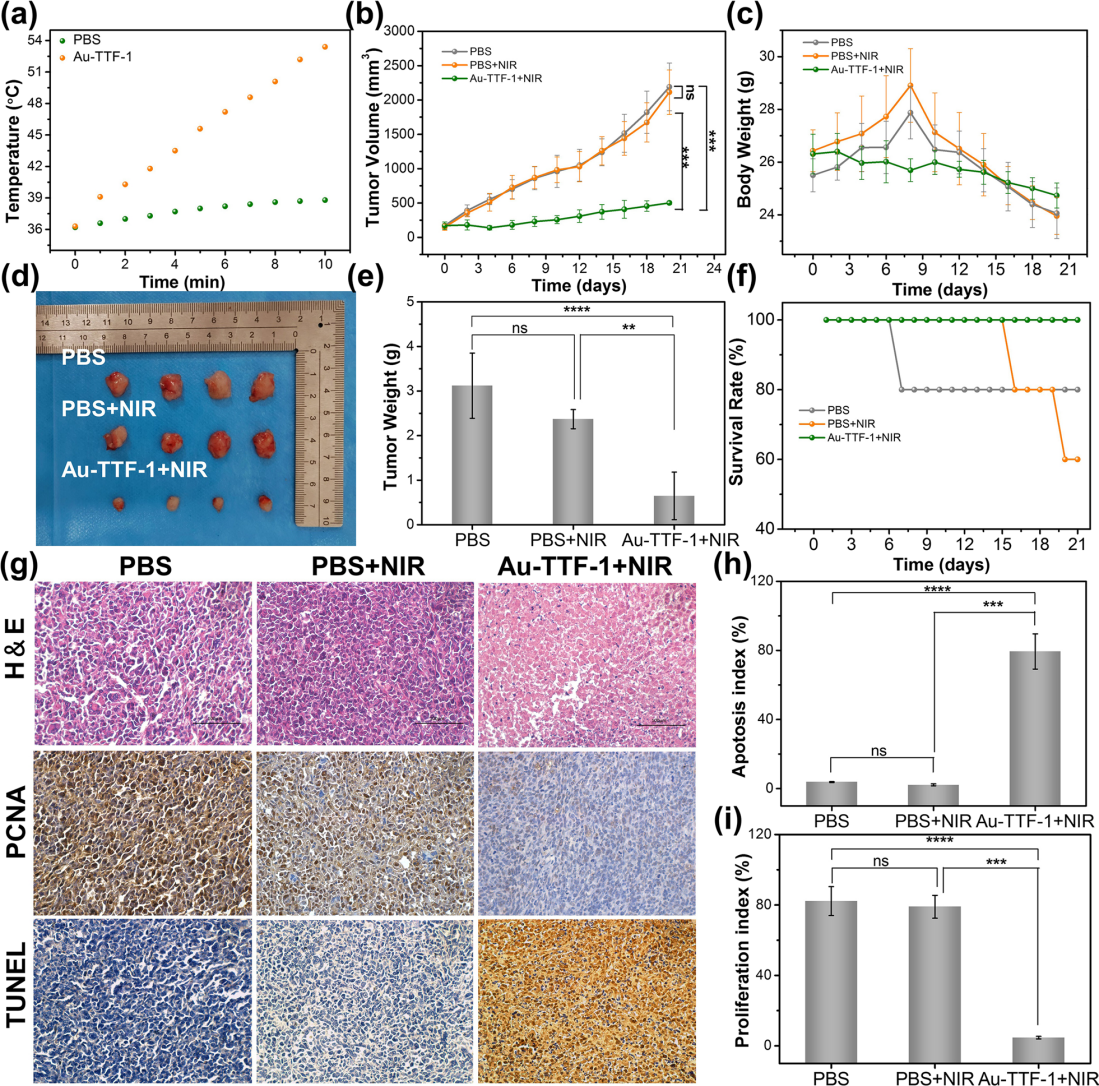
**a** Photothermal heating curves of AuNCs and Au-TTF-1 upon 808 nm laser irradiation. **b** Relative tumor volume growth curves of various groups in 21 days. **c** Body weight change of each group in 21 days of treatment. **d** Gross images of the excised tumors after 21 days. **e** Average tumor weight of different groups in 21 days. **f** Survival rate of various groups in 21 days. **g** H&E, TUNEL, and PCNA staining of tumor tis-sues after 21 days of treatment. **h** The apoptosis index of experimental and control group. (i) The proliferation in-dex of experimental and control group (*p < 0.05 and ***p < 0.001)

Based on the excellent imaging and target anticancer performance of Au-TTF-1 in cell experiments, mice-bearing lung adenocarcinoma model was established. Au-TTF-1 was intravenous injected and evident fluorescent signals were observed in Fig. 7a. Tumor was light up within 1 h and achieved efficient enrichment lengthened out to 12 h. In Fig. 7b, Organo-FL showed that most of the nanoprobe was concentrated in tumor, and a small part was metabolized by the liver. FL imaging is more intuitive and easy to distinguish with the naked eyes, while CT imaging has the characteristics of high resolution, fast imaging speed, and 3D picture reconstruction. In Fig. 7c, the tumor site showed an obvious CT imaging effect after injection for 1 h. Further, Au-TTF-1 not only possessed dual-mode tumor target imaging characteristics, but also exhibited imaging-guided excellent PTT ability. In Fig. 7d and Fig. 8a, IR images of tumors were acquired at various time points during 600 s, the tumor temperatures in the experimental group were remarkably higher (53.4 °C) than those in the control group (38.8 °C). According to the reports, this temperature was able to kill cancer cells by melting the membrane immediately and causing protein denaturation, which was harmful to tumor tissues, while being relatively safe to normal tissues. In addition, the temperature increase was consistent with the results of in vitro experiments, indicating the stability of Au-TTF-1 in vivo once again.

The in vivo investigation of lung adenocarcinoma inhibition was conducted by treating with Au-TTF-1 therapeutic agents combined with NIR irradiation, and there were 21 days for the observation of the mice variation. As shown in Fig. 8b, Au-TTF-1 + NIR had a significant inhibition of tumor growth compared with control groups in 21 days. Besides, the body weight of the mice showed an obvious decrease in the control groups (Fig. 8c), while Au-TTF-1 had a therapeutic effect in improving the survival experience of mice. After treatment, the subcutaneous tumor tissues were stripped. The photothermal therapy effect of the Au-TTF-1 group achieved more intuitive suppression results in Fig. 8d. The tumor weight in Fig. 8e was consistent with tumor volume evaluation. In addition, they increased the survival rate in Fig. 8f. In Fig. 8g, H & E staining of tumor tissues indicated that no obvious change of the morphology occurred in the control and Au-TTF-1 groups, while there was necrotic tissue around the tumor in the Au-TTF-1 + NIR group, owing to the hyperthermia derived from Au-TTF-1. Subsequently, the apoptosis and proliferation inhibition of the tumor cells among the various groups were investigated by TUNEL and PCNA assays. The results further confirmed that Au-TTF-1 could effectively induce apoptosis and inhibit the proliferation of tumor through PTT (Fig. 8h, i).

**Fig. 9:**
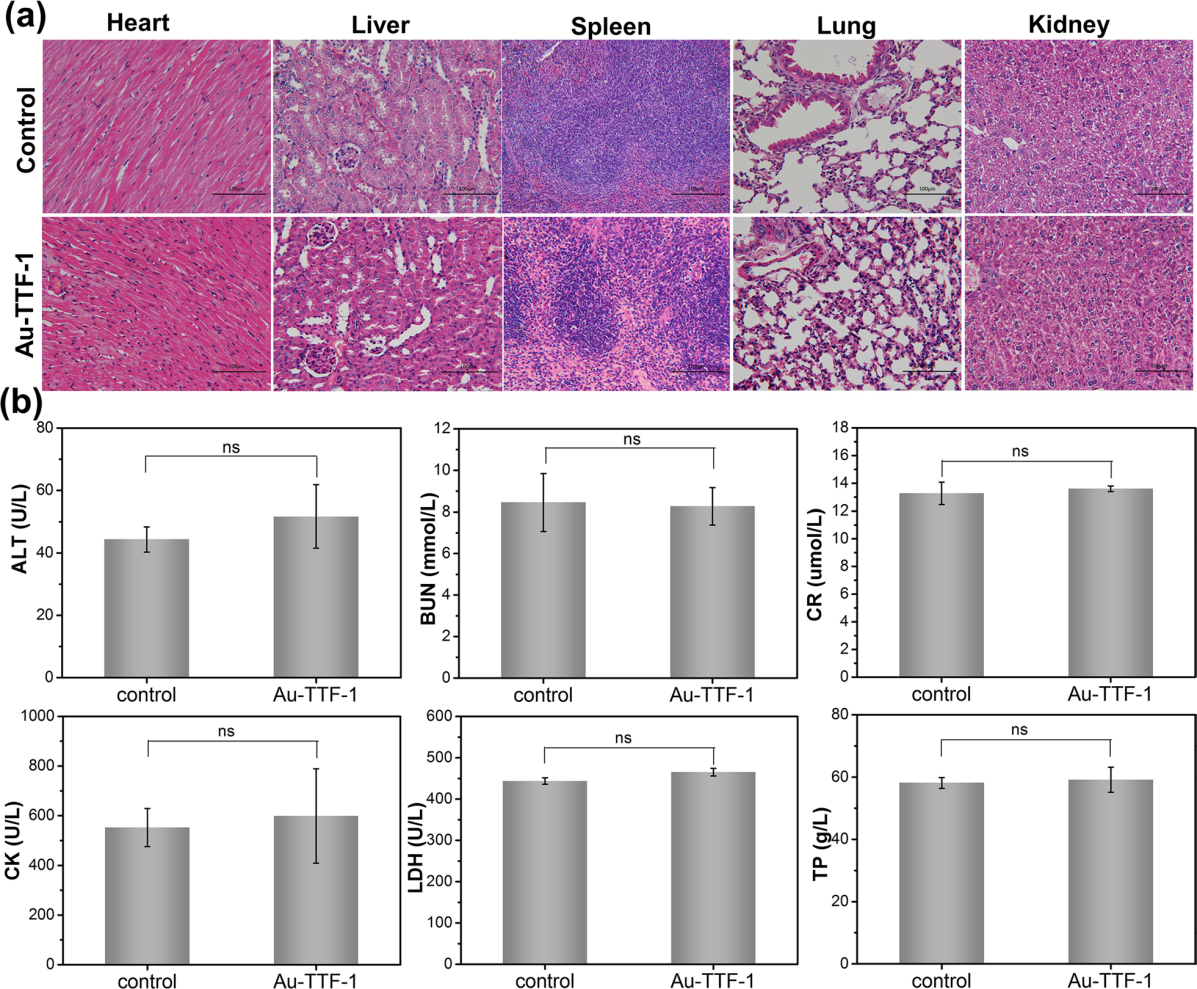
In vivo safety analysis. **a** H&E staining of histological analyses of main organs of each group after 21 days of treatment. **b** Serum biochemical parameters for cardiac function liver function and renal function

Safety is a crucial factor for nanoprobe. H&E staining analysis of major organs including the heart, liver, spleen, lung, and kidney in various groups was implemented to evaluate the biocompatibility of Au-TTF-1 (Fig. 9a). Conversely, no obvious organ damage or inflammation could be found. The serology detection for the function of the heart, liver and kidney in Fig. 9b, including creatine kinase (CK) and lactate dehydrogenase levels (LDH-L), alanine aminotransferase (ALT), and aspartate aminotransferase (AST), creatinine (CR), and blood urine nitrogen (BUN), also demonstrated that the Au-TTF-1 had no side effect after therapy. The above results showed that PTT therapy has satisfactory tumor killing ability and good application prospects.

## Conclusions

In this work, we designed and synthesized a multifunctional nanoprobe Au-TTF-1 and machine learning software-assisted detection system for lung adenocarcinoma. The uptake rate of Au-TTF-1 by lung adenocarcinoma cells was 3 times higher than lung epithelial cells, achieving effective target effect with bright red FL. The auxiliary system assisted in the diagnosis of lung adenocarcinoma through machine learning technology. Besides, Au-TTF-1 inhibited tumor growth through FL/CT imaging-guided photothermal therapy. Overall, our results demonstrate that Au-TTF-1 exhibited satisfactory results against lung adenocarcinoma, as it was able to precisely position the tumor before treatment, and provide imaging-guided PTT in a single nanoprobe.

### Supplementary Information

**Additional file 1: Figure. S1. **The hydrodynamic radius of AuNCs and Au-TTF-1.** Figure. S2.** **a** The optical stability of the fluorescent Au-TTF-1 in 21 days. **b** In NaCl solvent. **c** Under different pH range from 3 to 10. **d** Under UV lamp irradiation.** Figure. S3. **Flow cytometry analysis of cellular uptake of A549 cells, Beas-2B cells and H460 cells cultured with 300 μg/mL Au-Dsg-3 for 6 h. **Figure. S4.** Laser scanning confocal micrographs of A549 cells after culturing with Au-TTF-1 and dapi. **Video S1.** Demonstration procedure of software Lung adenocarcinoma auxiliary detection system.

## Data Availability

We encourage fellow scholars and the public to use, cite, and share data from this submission in compliance with copyright law, relevant laws and regulations. If you have any questions or requests regarding the use of the data, please contact corresponding author by email (Linquan@jlu.edu.cn).
