# Supplementary material for: “All in one” nanoprobe Au-TTF-1 for target FL/CT bioimaging, machine learning technology and imaging-guided photothermal therapy against lung adenocarcinoma
Source: J Nanobiotechnology. 2024 Jan 6;22:22. doi: 10.1186/s12951-023-02280-9 (PMC10770976; doi:10.1186/s12951-023-02280-9)
Supplement: Supplementary file 1 — Additional file 1: Figure. S1. The hydrodynamic radius of AuNCs and Au-TTF-1. Figure. S2. a The optical stability of the fluorescent Au-TTF-1 in 21 days. b In NaCl solvent. c Under different pH range from 3 to 10. d Under UV lamp irradiation. Figure. S3. Flow cytometry analysis of cellular uptake of A549 cells, Beas-2B cells and H460 cells cultured with 300 μg/mL Au-Dsg-3 for 6 h. Figure. S4. Laser scanning confocal micrographs of A549 cells after culturing with Au-TTF-1 and dapi. Video S1. Demonstration procedure of software Lung adenocarcinoma auxiliary detection system. [file 12951_2023_2280_MOESM1_ESM.docx]

**“All in one” nanoprobe Au-TTF-1 for target FL/CT bioimaging, machine learning technology and imaging-guided photothermal therapy against lung adenocarcinoma**

Zhe Yang^1, †^, Yujia Zhang ^2, †^, Lu Tang^3^, Xiao Yang^4^, Lei Song^3^, Chun Shen^4^, Andrei V. Zvyagin^5^, Yang Li ^2,*^, Bai Yang^1^, Quan Lin^1,*^

^1^ State Key Laboratory of Supramolecular Structure and Material, College of Chemistry, Jilin University, Changchun 130012, China.

^2^ Department of Respiratory Medicine, The First Hospital of Jilin University, Changchun 130021, China

^3^ Department of Breast, China-Japan Union Hospital of Jilin University, Changchun 130031, China

^4^ College of Computer Science and Technology Jilin University, Changchun 130012, China

^5^ Australian Research Council Centre of Excellence for Nanoscale Biophotonics, Macquarie University, Sydney, NSW 2109, Australia.

E-mail: [linquan@jlu.edu.cn](mailto:linquan@jlu.edu.cn); li_yang99@jlu.edu.cn

The PDF file includes:

Experimental Section

**Figure S1.** The hydrodynamic radius diameter of AuNCs and Au-TTF-1.

**Figure S2.** (a) The optical stability of the fluorescent Au-TTF-1 in 21 days. (b) In NaCl solvent. (c) Under different pH range from 3-10. (d) Under UV lamp irradiation.

**Video S1.** Demonstration procedure of software Lung adenocarcinoma auxiliary detection system.

**Experimental Section**

*Materials*

3-mercaptopropionic acid (MPA, 99%), branched polyethyleneimine (PEI, Mw=600 g/mol), chloroauric acid (HAuCl_4_) Tween-20 were purchased from Aldrich; 1-ethyl-(3-two Methylaminoethyl) carbodiimide hydrochloride (EDC), N-hydroxysuccinimide (NHS) were purchased from Aladdin Company; N, N-dimethylformamide (DMF) were purchased from Beijing Chemical Plant; acetone, chloroform, and hydrazine hydrate (N_2_H_4_·H_2_O, 85 wt%) were purchased from Sinopharm Chemical Reagent Co., Ltd. Thyroid Transcription Factor-1 (TTF-1) antibody was purchased from Abcam. Cell Counting Kit-8 (CCK-8) and Calcein-AM/Propidium Iodide (PI) were purchased from Beyotime Biotechnology. Hematoxylin-eosin (H&E) stains were purchased from Boster Biological Technology. Terminal deoxynucleotidyl transferase mediated dUTP nick-end labeling (TUNEL) was supplied by Elabscience. Dulbecco's modified eagle medium (DMEM) were supplied by Gibco. Proliferating cell nuclear antigen (PCNA) antibody was purchased from Affinity Biosciences. Anti-fluorescence attenuation mounting medium with DAPI was purchased from Solarbio. Life Sciences. All reagents were not been further purified before use. The water used in the whole experiment was secondary deionized water (18.2 MΩ×cm^-1^). Human lung adenocarcinoma cells (A549) and human normal lung epithelial cells (Beas-2B) were obtained from the Respiratory Laboratory of the First Hospital of Jilin University (Jilin, China).

*Instruments*

Transmission electron microscopy (TEM) photos were measured by JEOL TECNAI F20 transmission electron microscope at an operating voltage of 200 kV. Optical properties were measured by Shimadzu RF-5301 PC fluorescence spectrometer. Ultraviolet-visible absorption spectrum was measured by Lambad 800 ultraviolet-visible spectrophotometer. X-ray photoelectron spectroscopy was measured by VG ESCALAB MKII spectrometer. Zeta potential was measured by Zetasizer Nano ZS particle sizer. Laser scanning confocal microscope photograph was measured by Olympus Fluoview FV1000 confocal microscope. Cell activity data was measured by a microplate reader BioTek, Winooski, VT, USA. Fluorescence intensity analysis was performed by flow cytometry using a BD FACSVerse and FlowJo_V10 cytometer. Blood chemical analysis was performed using Smart Biochemical Ten-item kit (Chengdu Pulifeng Biotechnology Co., LTD.) for serum biochemical analysis. In vivo images were captured by PerkinElmer IVIS Lumina LT III. The temperature and images in different conditions were monitored by an infrared thermal imaging camera (FLIR T420).

*Preparation of ligand (SH-PEI):*

Dissolve 38 mg NHS and 64 mg EDC in 40 mL DMF, 300 μL MPA was then added into the system under continuous stirring for 30 min. Add 1 mL (350 mg/mL) PEI to the solution slowly and keep the system at room temperature under the protection of N_2_ atmosphere for 48 h.The product was concentrated to ~ 5 mL by distillation, and then precipitant was added (a mixed solvent of acetone and chloroform) to it. Centrifuge the mixture at 8000 rpm for 8 min, and collect the precipitate and redissolve it in 5 mL H_2_O. The product was stored at 4 ˚C for later use.

*Preparation of AuNCs*

250 μL prepared ligand was added to 5 mL H_2_O, and then 250 μL HAuCl_4_ (50 mM) was added. Finally, 300 μL N_2_H_4_·H_2_O was added to the mixture as a reducing agent. The solution was stirred at 80 ˚C for 4 h, and the product was dialyzed for 8 h to remove impurities. The outcome AuNCs aqueous solution was freeze-dried and stored at room temperature for later use.

*Cell culture and in vitro Biocompatibility Analysis*

All cells were cultured at 37°C in a 5% CO_2_ incubator. The medium was prepared with DMEM, 10% fetal bovine serum, 1% HEPES solution and 1% dual antibody solution. For in vitro cytotoxicity test, A549 and Beas-2B cells were cultured in 96-well plates, and Kit-8 (CCK-8) assay was performed the next day when the cell count was 8×10^3^. The medium was removed and fresh medium with different concentrations of 50 μg/mL, 100 μg/mL, 200 μg/mL and 300 μg/mL Au-TTF-1 was added. The mixture of target cells and AuNCs were incubated at 37 ° C for 6 h, 12 h and 24 h. Then, the medium was removed, washed twice with PBS, and then incubated in fresh CCK-8 solution medium for 1 h at 37 °C. Three separate biological experiments were conducted.

**Fig. S1.** The hydrodynamic radius of AuNCs and Au-TTF-1.


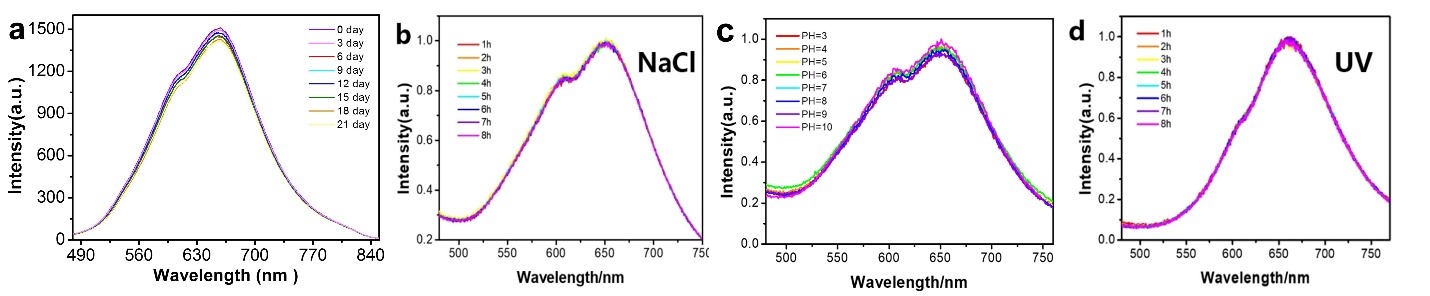


**Fig. S2.** (a) The optical stability of the fluorescent Au-TTF-1 in 21 days. (b) In NaCl solvent. (c) Under different pH range from 3-10. (d) Under UV lamp irradiation.

**Fig. S3.** Flow cytometry analysis of cellular uptake of A549 cells, Beas-2B cells and H460 cells cultured with 300 μg/mL Au-Dsg-3 for 6 h.


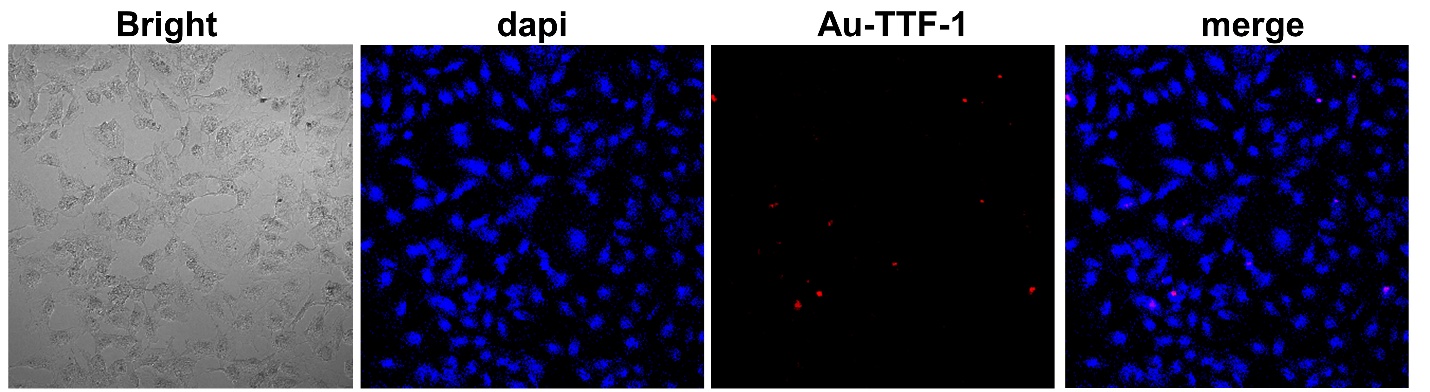


Fig. S4. Laser scanning confocal micrographs of A549 cells after culturing with Au-TTF-1 and dapi.

**Video S1.** Demonstration procedure of software Lung adenocarcinoma auxiliary detection system.
